# Pre-Administration of *Saccharomyces boulardii*-Derived Postbiotics Effectively Prevents Dextran Sulfate Sodium-Induced Colitis in Mice

**DOI:** 10.3390/foods14071109

**Published:** 2025-03-23

**Authors:** Yuxin Jin, Xinge Xu, Kunlun Huang, Zhihong Liang

**Affiliations:** 1 College of Food Science and Nutritional Engineering, China Agricultural University, Beijing 100083, China; j17860722880@163.com (Y.J.); s20223061103@cau.edu.cn (X.X.); hkl009@163.com (K.H.); 2 Beijing Laboratory for Food Quality and Safety, College of Food Science and Nutritional Engineering, China Agricultural University, Beijing 100083, China

**Keywords:** *Saccharomyces boulardii*, postbiotics, probiotic, ulcerative colitis, gut microbiota, inflammatory

## Abstract

Ulcerative colitis (UC) is effectively alleviated by *Saccharomyces boulardii* (*S. boulardii*), an important probiotic. Postbiotics, defined as beneficial non-viable microorganisms and/or their components, can potentially improve gut health. In this study, we utilized *S. boulardii* to prepare postbiotics via freeze-drying and spray-drying methods, characterized the resulting postbiotics, and investigated their efficacy and underlying mechanisms in preventing UC. In a mouse model of UC induced by dextran sulfate sodium (DSS), we found that prevention with two forms of *S. boulardii* postbiotics alleviated colitis symptoms triggered by DSS, mitigated colon tissue damage, maintained the distribution of intestinal occludin and ZO-1 proteins, and suppressed the secretion and expression of TNF-α, IL-1β, and IL-6 in serum and colon tissues. Additionally, *S. boulardii* postbiotics mitigated dysbiosis by modulating gut microbiota composition, including the balance between Bacteroidota and Firmicutes (F/B), as well as the levels of *Akkermansia*, *Muribaculaceae*, *Dubosiella*, and *Turicibacter*. In conclusion, as a novel biotherapeutic agent, *S. boulardii* postbiotics effectively prevent DSS-induced UC in mice. Compared to live *S. boulardii*, postbiotics may hold greater potential for UC prevention.

## 1. Introduction

Ulcerative colitis (UC) is one of the two types of inflammatory bowel disease (IBD), affects around five million people globally as of 2023, with incidence rates steadily rising [1,2]. UC primarily impacts the colon and rectum, causing continuous inflammation that can spread to the entire colon in severe cases [3]. Current treatments, including corticosteroids, immunomodulators, biologics, and surgery, are limited by low remission rates, high relapse rates, and high costs, driving novel therapies [4,5]. Emerging research suggests that gut microbiota dysbiosis may be a key factor in UC pathogenesis [6,7,8], making probiotics a potential therapeutic option.

*Saccharomyces boulardii* (*S. boulardii*) is a probiotic that can exhibit functions such as anticancer, antimicrobial, antioxidant, and immune modulation in the body [9,10]. Since the 1950s, *S. boulardii* has been widely used to prevent and treat antibiotic-associated diarrhea. It has also shown some effectiveness in preliminary studies on patients with IBD [11]. Although *S. boulardii* has shown promising potential in early studies, it has also raised safety concerns, including the risk of fungal infections and interactions with gut bacteria such as *Escherichia coli* [12,13]. These concerns highlight the need for safer alternatives. In recent years, postbiotics have gained widespread attention as a substitute for probiotics. They refer to non-viable probiotics or their components, which can provide health benefits similar to those of probiotics without relying on live bacteria growth [14,15,16]. The efficacy of postbiotics stems from the metabolites in cell components or fermentation substrates, and the viability of microorganisms is not a necessary condition for exerting gut-protective effects. Therefore, postbiotics may represent a better and safer strategy for treating colitis [17]. The mechanisms of postbiotics in improving the gut microbiome and their anti-inflammatory potential are still under exploration, but existing studies have suggested that postbiotics can exert anti-inflammatory effects by modulating the gut microbiome, repairing the intestinal barrier function, and activating the host immune system [18,19]. Compared to traditional probiotics, postbiotics have the advantage of not relying on live bacterial growth and reproduction, thereby avoiding potential risks such as microbial imbalance or overgrowth in unfavorable intestinal environments. Additionally, postbiotics may provide sustained anti-inflammatory effects due to the direct action of their metabolites and cell components.

Our team is dedicated to studying postbiotics derived from *S. boulardii* probiotics. Previous research has already confirmed that timely supplementation with *S. boulardii* postbiotics can significantly inhibit disease progression of UC in mice [20]. In this study, we adopted a different intervention strategy by starting postbiotic treatment before dextran sulfate sodium (DSS) induction to evaluate its preventive effect on colitis. This design more clearly emphasizes the potential of postbiotics in disease prevention rather than merely alleviating existing conditions. Through this prospective intervention, our study provides more clinically meaningful support for the application of postbiotics in colitis prevention. This study characterizes the structure of *S. boulardii* postbiotics prepared using different methods. We investigated the impact of *S. boulardii* probiotics and postbiotics obtained from two different preparation methods on the gut microbiome of DSS-induced colitis mice, as well as their effects on preventing inflammatory responses. Through this research, we aim to elucidate the potential of postbiotics in preventing colitis and establish their status as a novel biotherapeutic agent.

## 2. Materials and Methods

### 2.1. Major Reagents

Rabbit ZO-1 polyclonal antibody (61-7300), rabbit occludin polyclonal antibody (40-4700), and goat anti-rabbit IgG (H + L) secondary antibody, HRP (31460) (Thermo Fisher Scientific, Waltham, MA, USA). Mouse IL-6 (SEKM-0007), IL-1β (SEKM-0002), and TNF-α (SEKM-0034) ELISA Kits (Solarbio Science & Technology Co., Ltd., Beijing, China). TransZol Up Plus RNA kit (ER501-01), and TransScript One-Step gDNA Removal and cDNA Synthesis SuperMix (AT311-02) (TransGen Biotech Co., Ltd., Beijing, China). SuperReal PreMix Plus (SYBR Green) (FP205-01) (Tiangen Biotech Co., Ltd., Beijing, China).

### 2.2. Preparation of Postbiotics

The *S. boulardii* used in the study is the purchased strain (CCTCC NO.M 2012116) stored in the laboratory. The preparation methods for freeze-dried and spray-dried *S. boulardii* postbiotics were the same as those previously described [20]. The dried postbiotics obtained by both methods were ground into powder and stored. In this experimental method, the concentration of *S. boulardii* in the stationary phase was 10^8^ CFU/mL. At this concentration, the *S. boulardii* suspension was inactivated at 121 °C for 20 min, then aliquoted and used to prepare postbiotics.

### 2.3. Characterization of Postbiotics

The morphology of *S. boulardii* and two forms of postbiotics were visualized by scanning electron microscopy (SEM, Hitachi Co., Ltd., Beijing, China).

### 2.4. Animals

C57BL/6J male mice, 7 weeks old, 20 ± 2 g, were sourced from Beijing Vital River Laboratory Animal Technology Co., Ltd. (SCXK (Beijing, China) 2021-0006). All experimental mice were grouped and housed under specific pathogen-free (SPF) conditions. In this study, five mice were housed in each individually ventilated cage. The temperature was maintained at 24 ± 1 °C, the relative humidity was kept 40–70%, and the 12 h light/12 h dark cycle mimicked the natural circadian rhythm.

### 2.5. Animals Experiment

Fifty male C57BL/6J mice were randomly assigned into five groups (n = 10) after a one-week acclimation: healthy control (Control), DSS-induced colitis model (DSS), *S. boulardii* preventive gavage (BLD), freeze-dried *S. boulardii* postbiotics preventive gavage (DBLD), and spray-dried *S. boulardii* postbiotics preventive gavage (PBLD). Postbiotic powders were dissolved in PBS to achieve a gavage concentration of 2 g/L per mouse. For 21 days, each group was orally gavaged daily with 200 μL of their respective solutions (PBS, PBS, *S. boulardii*, freeze-dried postbiotics, or spray-dried postbiotics), with no restrictions on normal food and water intake. On days 22–28, the DSS, BLD, DBLD, and PBLD groups received 2% DSS in drinking water, refreshed daily. The changes in body weight and Disease Activity Index (DAI) during the DSS induction phase (days 22–28) were recorded, with the DAI scoring criteria provided in Supplementary Table S1. On day 29, the mice were euthanized by cervical dislocation. Organs (liver, kidneys, and spleen) were dissected and weighed to calculate organ indices (organ weight/body weight × 100%).

### 2.6. Histopathological Analysis and Immunohistochemical Analysis

After measuring the length of the colon tissue, it was rinsed 2–3 times. A 50 mm segment of colon tissue located 1–2 cm from the anus was collected and fixed in 4% paraformaldehyde. The fixed tissue was embedded in paraffin, sectioned, and then stained with hematoxylin and eosin (H&E). The stained sections were observed using a SUNNY EX20 biological microscope (Sunny Instruments Co., Ltd., Ningbo, China). For another part of the colonic sections used for immunohistochemistry (IHC), the tissue sections were deparaffinized, subjected to gradient rehydration, and then thoroughly washed with deionized water. The sections were incubated overnight at 65 °C in citrate buffer for antigen retrieval. After cooling, the sections were washed with PBS, followed by the addition of 50 μL 3% hydrogen peroxide solution for 10 min, and washed again with PBS. Blocking with 50 μL 5% BSA for 30 min, then incubated with 50 μL of 1:200 diluted ZO-1 and occludin primary antibodies overnight at 4 °C. The sections were incubated with 50 μL of 1:500 secondary antibody for 30 min, washed with PBS, and stained with Hematoxylin for 2 min. Finally, the sections were observed under a microscope with quantitative analysis using ImageJ (V1.8.0). IHC score = (staining intensity score × positive cells %). Staining intensity was scored on a 4-point scale: negative (0), weak positive (1), moderate positive (2), and strong positive (3). Positive cell % was classified into 5 levels: <10% (1), 10–25% (2), 26–50% (3), 51–75% (4), and >75% (5). All scores were normalized to the Control group.

### 2.7. Detection of Inflammatory Factor Levels

After standing, the mouse blood samples were centrifuged at 4 °C for one time (3500 rpm, 15 min). Following the manufacturer’s instructions, TNF-α, IL-1β, and IL-6 levels in the *serum* were determined with corresponding ELISA kits. Colon RNA was extracted with the TransZol Up Plus RNA kit and converted into cDNA using a reverse transcription enzyme kit. The resulting cDNA was analyzed by real-time quantitative PCR with SuperReal PreMix Plus (SYBR Green). *TNF-α*, *IL-1β*, and *IL-6* mRNA expression levels in colon tissue were determined using the 2^−ΔΔCt^ method, normalized to GAPDH. The primer sequences are listed in Supplementary Table S2.

### 2.8. Analysis of Gut Microbiota

We extracted the microbial genomic DNA, amplified the V3–V4 region of the bacterial 16S rRNA gene, and sequenced on the NovaSeq platform (Illumina, San Diego, CA, USA). The raw data were filtered and analyzed using QIIME (v1.9.1). OTU clustering was performed with UPARSE v7.0.1001 at a 97% identity threshold. Based on the sequencing results, linear discriminant analysis (LDA) was conducted, and Linear Discriminant Analysis Effect Size (LEfSe) was used to compare the effect sizes (LDA score > 4).

### 2.9. Statistical Analysis

Data are expressed as means ± SD deviation and analyzed using the statistical software GraphPad Prism 9.0 (GraphPad Software Inc., San Diego, CA, USA). One-way ANOVA was employed to assess differences among groups, with statistical significance set at *p* < 0.05.

## 3. Results

### 3.1. Morphological Characterization of the Postbiotic of S. boulardii

**Figure 1:**
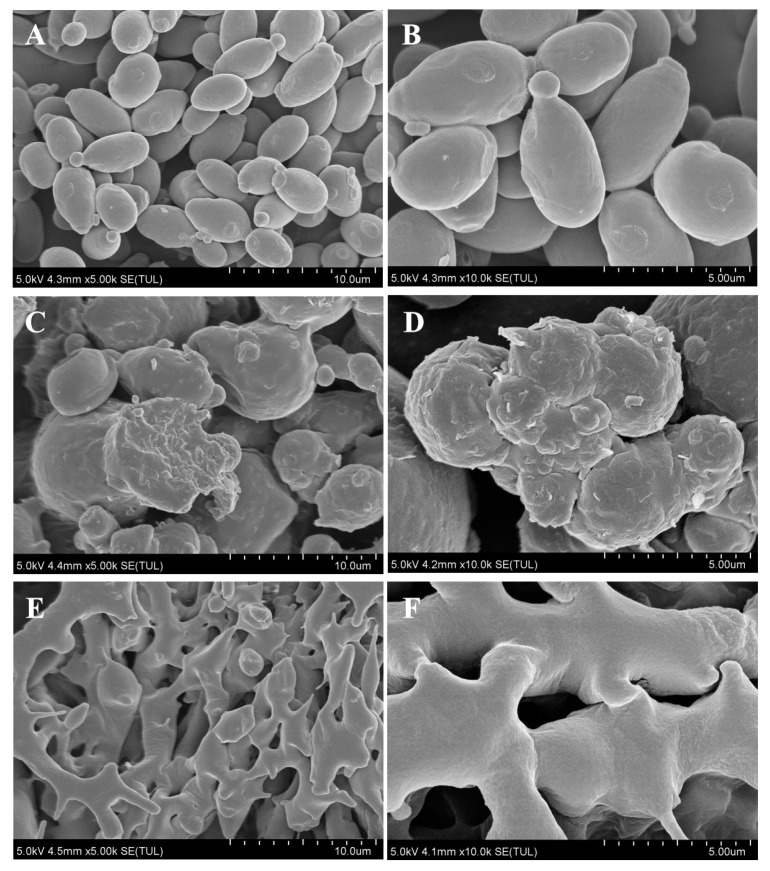
Morphological changes of postbiotics of *S. boulardii*. SEM observation of *S. boulardii* (**A**) 5 k× magnification, (**B**) 10 k× magnification. SEM observation of spray-dried postbiotics of *S. boulardii* (**C**) 5 k× magnification, (**D**) 10 k× magnification. SEM observation of freeze-dried postbiotics of *S. boulardii* (**E**) 5 k× magnification, (**F**) 10 k× magnification.

Both spray-drying and freeze-drying methods were employed to prepare postbiotics, resulting in powdered samples (Figure S1). Scanning electron microscopy (SEM) magnification revealed the detailed structure of the fungal cells. The Control group sample of *S. boulardii* exhibited intact and smooth-surfaced fungicide, displaying a normal spherical shape with evenly distributed surface texture (Figure 1A). Conversely, the postbiotics prepared by spray-drying showed collapsed fungicide (Figure 1B), characterized by sunken or wrinkled surfaces, leakage of contents, and noticeable morphological changes in buds, indicating significant damage to cell walls and membranes. In contrast, the postbiotics prepared by freeze-drying displayed signs of freezing (Figure 1C), with parent cells being extensively disrupted while some budding cells remained. Deconstruction and association phenomena occurred following bacterial breakage, resulting in damaged surface structures, and individual intact parent cells were no longer discernible.

### 3.2. The Intervention of S. boulardii Postbiotics Attenuated DSS-Induced Changes in Growth Performance

**Figure 2:**
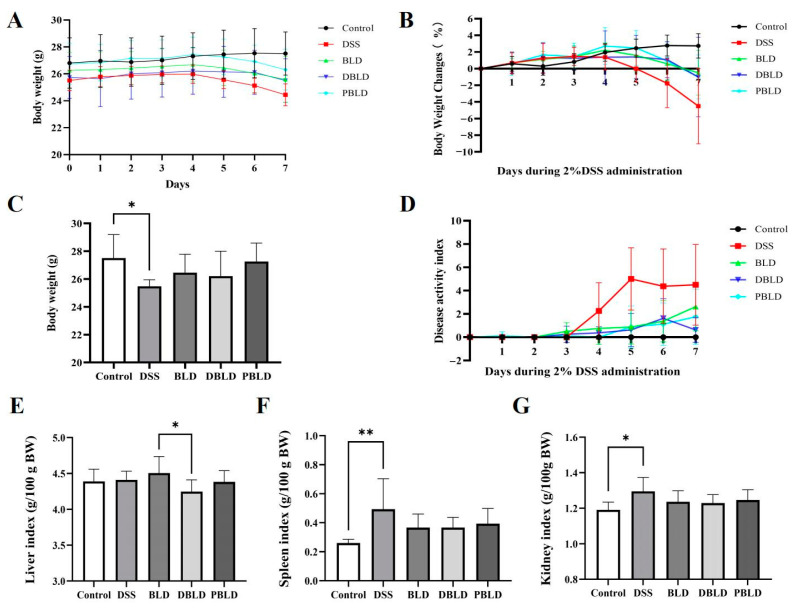
Prevention effect of *S. boulardii* and its postbiotics on body weight, DAI, and organ indices in DSS-induced colitis mice. (**A**) Body weight. (**B**) Body weight change. (**C**) Final weight. (**D**) DAI index. (**E**) Liver index. (**F**) Spleen index. (**G**) Kidney index. Note: Data are expressed as mean ± SD (n = 8). Statistical differences are marked as follows: * *p* < 0.05, ** *p* < 0.01.

Body weight and DAI during the DSS induction phase are important indicators for evaluating the efficacy of the three preventive treatment groups. The results showed that compared to the Control group, the body weight of the DSS group mice showed an overall upward trend in the first three weeks but started to decline on the fifth day of DSS induction, with a significant decrease in final body weight (*p* < 0.05) (Figure 2A–C). Mice in the BLD, DBLD, and PBLD groups also experienced some weight loss during DSS induction, but their final body weight did not significantly differ from the Control group (Figure 2A–C). Regarding the DAI, on the third day after DSS induction, the DAI scores began to increase in all groups, with the DSS group showing the most significant increase (Figure 2D). The above results indicate that a three-week pre-administration of *S. boulardii* and its postbiotics could effectively delay the onset of DSS-induced inflammation.

In most inflammatory responses, the spleen undergoes immune activation and subsequently increases in size [21]. Our results confirmed this finding. Compared to the Control group, the spleen index of mice in the DSS group significantly increased (*p* < 0.05), while preventive administration of *S. boulardii* and its postbiotics prevented this effect, with no significant difference in spleen index compared to the Control group (*p* > 0.05) (Figure 2F). The treatment with DSS, BLD, DBLD, and PBLD did not significantly affect the liver and kidney indices compared to the Control group (*p* > 0.05) (Figure 2G). It is worth noting that there was a difference in liver index between the BLD and DBLD groups, with the BLD group showing significantly higher liver index than the DBLD group (*p* < 0.05) (Figure 2E).

### 3.3. S. boulardii Postbiotics Mitigated DSS-Induced Alterations in Colon Tissue Structure

**Figure 3:**
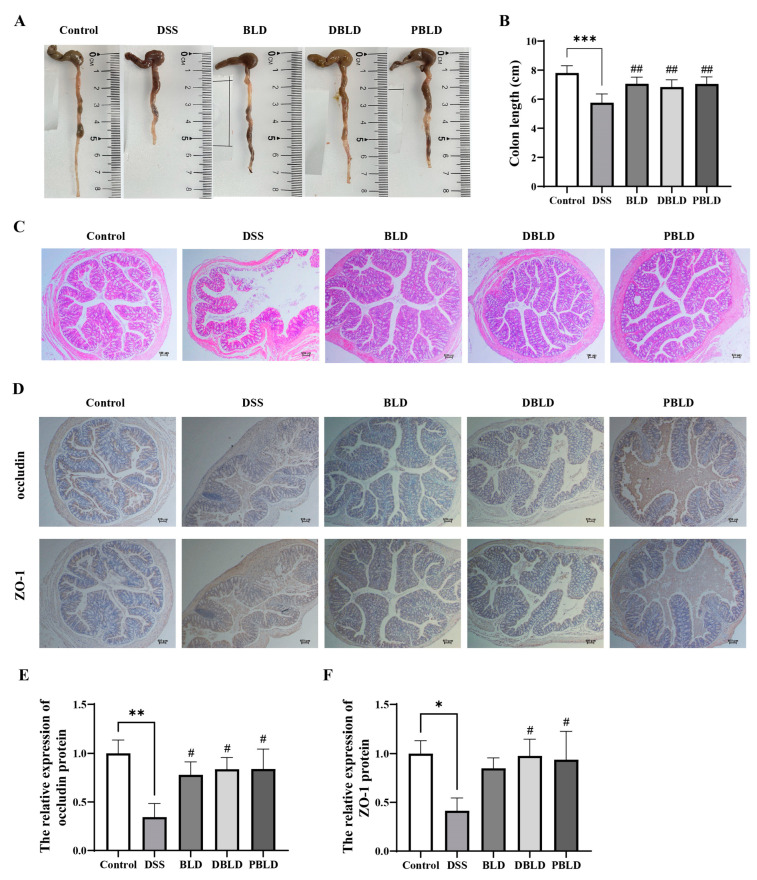
Prevention effect of *S. boulardii* and its postbiotics on colon length and tissue morphology in mice with DSS-induced colitis. (**A**) Mouse colon images. (**B**) Colon length. (**C**) Colon H&E staining images (40× magnification). (**D**) Colon IHC staining images (40× magnification). (**E**) Relative expression of the occludin protein. (**F**) Relative expression of ZO-1 protein. Note: Data are expressed as mean ± SD (n = 8). Statistical differences are marked as follows: * *p* < 0.05, ** *p* < 0.01, and *** *p* < 0.001 vs. Control; # *p* < 0.05 and ## *p* < 0.01 vs. DSS.

Colon length reduction is a characteristic feature of DSS-induced colitis [22]. The DSS group showed significantly shorter colons than other groups (*p* < 0.01) (Figure 3A). After preventive intervention with *S. boulardii* and its postbiotics, the BLD, DBLD, and PBLD groups significantly alleviated DSS-induced colon shortening (*p* < 0.01) (Figure 3B). The tissue staining results show severe colon damage in the DSS group, with multiple crypt structures deformed or even disappearing and evident infiltration of inflammatory cells. In contrast, the BLD, DBLD, and PBLD groups exhibited improved colon morphology, with reduced inflammation and preserved tissue structure (Figure 3C). IHC results showed uniform and strong expression of occludin and ZO-1 in the Control group, indicating intact intestinal tight junctions. In the DSS group, staining was weakened and uneven, reflecting the disruption of tight junctions. The BLD, DBLD, and PBLD groups exhibited improved staining intensity and uniformity compared to the DSS group (Figure 3D). Among them, DBLD and PBLD significantly improved the content and distribution of both occludin and ZO-1 proteins in the colon (Figure 3E,F). These findings indicate that prophylactic administration of *S. boulardii* and its postbiotics could alleviate DSS-induced pathological damage in colon tissue and protect intestinal tight junctions.

### 3.4. S. boulardii Postbiotics Inhibit the Increase of Pro-Inflammatory Cytokines Induced by DSS

**Figure 4:**
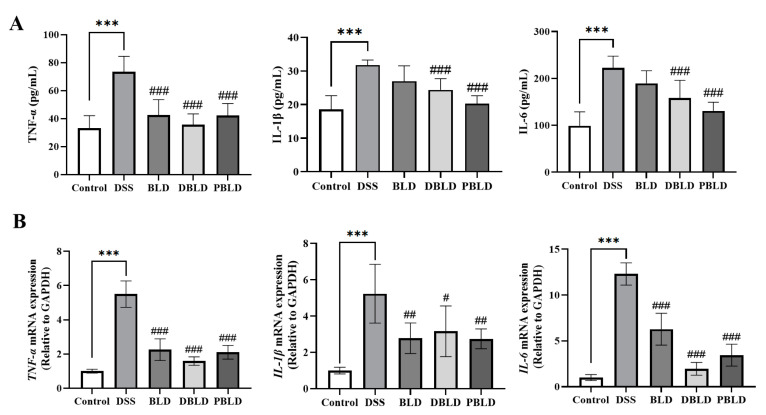
Prevention effect of *S. boulardii* and its postbiotis on the prevention of serum and colon inflammatory factors in mice with DSS-induced colitis. (**A**) Levels of TNF-α, IL-1β, and IL-6 in the serum. (**B**) Gene expression levels of *TNF-α*, *IL-1β*, and *IL-6* in the colon. Note: Data are expressed as mean ± SD (n = 8). Statistical differences are marked as follows: *** *p* < 0.001 vs. Control; # *p* < 0.05, ## *p* < 0.01, and ### *p* < 0.001 vs. DSS.

We performed measurements of inflammatory cytokine levels in the serum (Figure 4A). The results indicated that the serum levels of pro-inflammatory cytokines TNF-α, IL-1β, and IL-6 of mice from the DSS group were notably increased (*p* < 0.001). Compared to the DSS group, the BLD group exhibited a significant decrease in TNF-α levels (*p* < 0.01), while the levels of IL-1β and IL-6 remained significantly higher than those in the Control group (*p* < 0.001). The two postbiotics’ intervention effects were more pronounced than live *S. boulardii*. The DBLD and PBLD groups showed significant reductions in TNF-α, IL-1β, and IL-6 levels in the serum compared to the DSS group (*p* < 0.01). We also examined the mRNA expression of colonic inflammatory cytokines across the different groups of mice (Figure 4B). The DSS group showed a significant increase in the gene expression levels of *TNF-α*, *IL-1β*, and *IL-6* in the colon (*p* < 0.001), which was consistent with the serum findings, indicating a strong correlation between DSS-induced colitis and heightened cytokine expression in the colon. In contrast, all preventive interventions in the groups significantly reversed the elevated expression levels of inflammation-related genes (*p* < 0.05). These gene expression results align with serum immune responses and highlight the superior preventive effects of postbiotics.

### 3.5. S. boulardii Postbiotics Regulated the Diversity of the Gut Microbiota

**Figure 5:**
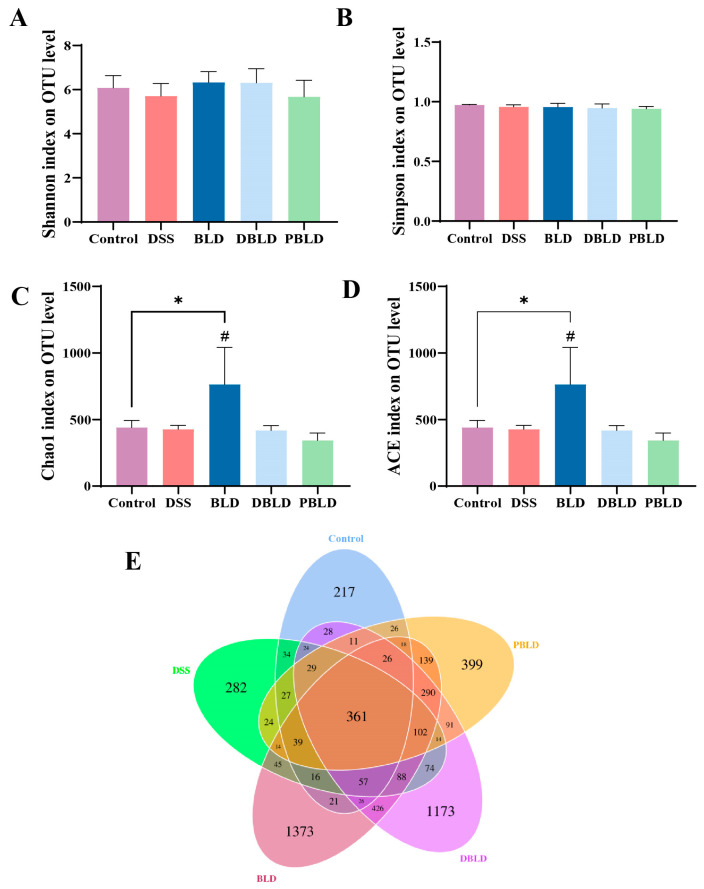
The influence of *S. boulardii* and its postbiotics on the diversity of gut microbiota. (**A**) Shannon index. (**B**) Simpson index. (**C**) Chao1 index. (**D**) ACE index. (**E**) Venn diagram. Note: Data are expressed as mean ± SD (n = 6). Statistical differences are marked as follows: * *p* < 0.05 vs. Control; # *p* < 0.05 vs. DSS.

α-diversity of cecal microbiota in different groups was evaluated using the Shannon index, Simpson index, Chao1 index, and ACE index. The results showed that DSS induction and all preventive groups did not significantly alter the Shannon index and Simpson index of the cecal contents in colitis mice (Figure 5A,B). For the Chao1 index and ACE index, the BLD group exhibited a significant increase compared to the Control and DSS groups (*p* < 0.05), indicating that prophylactic gavage with *S. boulardii* probiotics significantly affected the species richness of cecal microbiota (Figure 5C,D). DSS induction and the two postbiotic interventions had no significant impact on the α-diversity of cecal contents in mice. The Venn diagram illustrates the overlap and unique OTUs across the different sample groups. Mice treated with *S. boulardii* and its postbiotics had a significantly higher number of specific OTUs compared to the Control and DSS groups (Figure 5E). Among the treatment groups, mice pre-treated with *S. boulardii* had the highest total number of OTUs and specific OTUs. This is consistent with the results reflected by the Chao1 index and ACE index.

### 3.6. S. boulardii Postbiotics Altered the Composition of the Gut Microbiota

To investigate the specific effects of prophylactic gavage with *S. boulardii* and its postbiotics on the gut microbiota, as well as potential differences between different postbiotics, we compared the composition and relative abundance of the gut microbiota in each group of mice.

**Figure 6:**
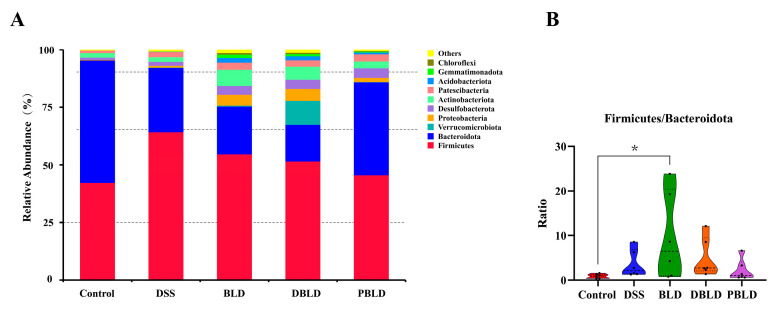
The influence of *S. boulardii* and its postbiotics on the phylum-level composition of gut microbiota. (**A**) Phylum-level composition of gut microbiota. (**B**) The balance between Firmicutes and Bacteroidetes. Note: Data are expressed as mean ± SD (n = 6). Statistical differences are marked as follows: * *p* < 0.05.

At the phylum level, the main components of the mouse intestinal flora were Firmicutes, Bacteroidota, Verrucomicrobiota, and Proteobacteria. Compared to the Control group, the DSS group showed an increased relative abundance of Firmicutes and a decreased relative abundance of Bacteroidota. Moreover, the prophylactic gavage of *S. boulardii* and its postbiotic could reverse the increase in the relative abundance of Firmicutes. Notably, the composition of the gut microbiota at the phylum level in the PBLD group was closest to that of the Control group (Figure 6A). Furthermore, the balance between Firmicutes and Bacteroidota (F/B) was analyzed (Figure 6B). The BLD group had a significantly higher F/B ratio (*p* < 0.05), while no significant differences were observed among the other groups. Furthermore, alongside Firmicutes and Bacteroidota, Verrucomicrobiota and Proteobacteria were also two important phyla in the mouse gut microbiota. The results indicate that the administration of freeze-dried postbiotics significantly increased the relative abundance of Verrucomicrobiota, showing significant differences compared to the other groups (Figure S2). Regarding Proteobacteria, although the administration of *S. boulardii* and spray-dried postbiotics caused considerable variability in the abundance of Proteobacteria, no significant differences were observed between the groups (Figure S3). The PBLD group showed an F/B, along with the abundance of Verrucomicrobiota and Proteobacteria, most similar to the Control group, suggesting that prophylactic gavage of spray-dried postbiotics could maximally reverse the phylum-level changes induced by DSS.

**Figure 7:**
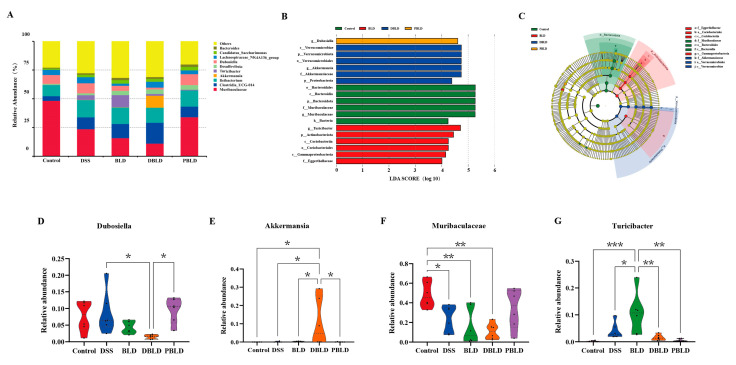
The influence of *S. boulardii* and its postbiotics on the genus-level composition of gut microbiota. (**A**) Genus-level composition of gut microbiota. (**B**) Indicator bacteria with LDA scores of >4 in five groups. (**C**) LEfSe Cladogram. (**D**) Differences in relative abundances of *Dubosiella* among the groups. (**E**) Differences in relative abundances of *Akkermansia* among the groups. (**F**) Differences in relative abundances of *Muribaculaceae* among the groups. (**G**) Differences in relative abundances of *Turicibacter* among the groups. Note: Data are expressed as mean ± SD (n = 6). Statistical differences are marked as follows: * *p* < 0.05, ** *p* < 0.01, and *** *p* < 0.001.

A genus-level analysis of gut microbiota abundance revealed significant differences across the groups. The gut microbiota of the Control group of mice was primarily composed of *Muribaculaceae* (48.31%), *Ileibacterium* (9.55%), *Dubosiella* (7.63%), *Lachnospiraceae*_NK4A136_group (4.53%), and *Clostridia* UCG-014 (4.08%) (Figure 7A). LEfSe analysis further highlighted distinct microbial enrichments in each group. Specifically, *Dubosiella* was enriched in the PBLD group. The Verrucomicrobiota, Proteobacteria, Verrucomicrobiae, Verrucomicrobiales, Akkermansiaceae, and *Akkermansia* were enriched in the DBLD group. The Bacteria, Bacteroidota, Bacteroidia, Bacteroidales, Muribaculaceae, and *Muribaculaceae* were enriched in the Control group. The Actinobacteriota, Coriobacteriia, Gammaproteobacteria, Coriobacteriales, Eggerthellaceae, and *Turicibacter* were enriched in the BLD group (Figure 7B,C). The LEfSe Cladogram plays a key role in visualizing the phylogenetic relationships and hierarchical structure of bacterial taxa. Using node colors, it allows for a quick identification of the specific groups with higher abundance of differential bacteria, corresponding with the LDA scores, and provides a clear depiction of the intergroup distribution of these bacteria. Additionally, the branching structure of the LEfSe Cladogram highlights the evolutionary relationships between differential bacteria, which cannot be captured by LDA scores alone.

Further analysis of key bacterial genera revealed that the relative abundance of *Dubosiella* decreased significantly in the DBLD group compared to the BLD and PBLD groups (*p* < 0.05). The relative abundance of *Akkermansia* increased significantly in the DBLD group, showing significant differences compared to the Control, DSS, BLD, and DBLD groups (*p* < 0.05). The relative abundance of *Muribaculaceae* decreased significantly in both the BLD and DBLD groups, with highly significant differences compared to the Control group (*p* < 0.01). The relative abundance of *Turicibacter* in the BLD group was significantly higher than that in the DSS group (*p* < 0.05) and highly significantly higher than that in the Control, DBLD, and PBLD groups (*p* < 0.01) (Figure 7D–G).

These results indicate that prophylactic gavage of *S. boulardii* and its freeze-dried postbiotics led to significant changes in the gut microbiota of mice. Specifically, these interventions counteract the changes induced by DSS, with the spray-dried postbiotics group showing a gut microbiota composition most similar to that of the Control group.

## 4. Discussion

As mentioned in the introduction, the occurrence of UC often leads to symptoms such as abdominal pain, diarrhea, and fatigue, which can significantly interfere with the patient’s normal life and daily activities. Currently, most medications used to treat UC can only temporarily alleviate symptoms and cannot cure the disease, and they are also costly [23]. Long-term illness can lead to emotional issues, malnutrition, and weight loss, further reducing the patient’s overall quality of life. Additionally, during treatment, frequent medical visits and medication use can impose psychological and financial burdens on the patient. This highlights the urgent need for safer, more effective preventive drugs. Research on *S. boulardii* has shown that it can improve UC through multiple mechanisms [24,25]. While the benefits of *S. boulardii* as a probiotic are well established, its postbiotics also show promise and deserve further exploration. This study used a DSS-induced colitis model to investigate the preventive and protective effects of *S. boulardii* and its postbiotics on UC. The findings provide a foundation for their potential use in UC prevention.

This study first characterized the cellular structure of *S. boulardii* and its postbiotics, filling the gap in the team’s previous research on microscopic structural understanding and providing crucial support for explaining the mechanisms of postbiotics at the cellular level. The observed damage and rupture of postbiotic cell structures indicated the release of various intracellular bioactive substances (such as proteins and polysaccharides) during preparation. These bioactive substances exert multiple beneficial effects on the intestine [26,27,28]. Therefore, despite the damage to cell structures, the functional components of the postbiotics were retained, which may explain why the therapeutic effects of the two postbiotics on colitis are comparable to those of probiotics. Unlike previous studies, this research employed a pre-intervention approach to explore the preventive effects of postbiotics before the onset of colitis. The findings suggest that the preventive intervention with *S. boulardii* and its postbiotics significantly alleviated weight loss and DAI in mice. They prevented colon shortening, tissue damage, and alterations in tight junction protein distribution. Postbiotics also inhibited the secretion and expression of pro-inflammatory cytokines in serum and the colon, stabilizing the gut microbiota. Therefore, regarding the anti-inflammatory effects observed in this study, postbiotics appear to have greater safety potential for the early prevention of UC.

Inflammation is a defensive response to pathogens, but when dysregulated, it exacerbates tissue damage, as seen in UC [29,30]. Inhibiting excessive inflammatory responses is thus a viable strategy for alleviating UC symptoms. Inflammatory cytokines are signaling molecules between cells and can mediate immune responses [31]. While this study found that inflammatory factors were excessively expressed in the model group mice, it also confirmed the significant inhibitory effects of postbiotics on inflammation. Notably, in the serum, the levels of IL-1β and IL-6 in the BLD group were similar to those in the DSS model group, raising concerns about potential immune stress caused by probiotics. In other related studies, similar instances have been observed where certain probiotic strains, under specific conditions, may trigger fluctuations in the body’s immune response, inducing a cell-mediated immune response that generates large amounts of pro-inflammatory cytokines, including IL-1β and IL-6 [32]. Another possible explanation for this phenomenon is that the preventive intervention with probiotics significantly reduced the mRNA expression of IL-1β and IL-6 in colon tissue, suggesting that probiotics can effectively alleviate local intestinal inflammation, possibly by modulating intestinal immune cells or improving the gut microbiota. However, the circulating levels of IL-1β and IL-6 in the serum did not show significant changes, indicating that the effects of probiotics are mainly concentrated in the local intestinal environment, with limited impact on systemic inflammation. This difference between local and systemic responses further suggests that probiotics primarily exert local anti-inflammatory effects in the treatment of colitis, rather than broad systemic immune modulation. In contrast, postbiotics, due to the release of active substances from disrupted bacterial cells, can directly enter the systemic circulation and produce systemic immune modulation, thereby exerting significant inhibitory effects on the circulating inflammatory factors. This mechanism may help explain the prominent effects of postbiotics in systemic inflammation.

Based on the anti-inflammatory effects observed in this study, postbiotics seem to offer higher safety in the early prevention of UC, positioning them as a more promising and ideal preventive approach. Nevertheless, the precise molecular mechanisms behind the anti-inflammatory effects of *S. boulardii* postbiotics remain unclear. Future studies may employ cutting-edge gene editing technologies to selectively knock out or overexpress genes potentially involved in the anti-inflammatory process. This would enable a deeper investigation into their influence on inflammation-related signaling pathways and shed light on the underlying molecular mechanisms of postbiotics’ anti-inflammatory action.

Over the past decade, numerous studies have highlighted the close relationship between characteristic alterations in the composition of the gut microbiota and the pathogenesis of IBD [33,34,35], emphasizing the dysregulation in the interaction between gut microbiota and the mucosal immune system as a crucial factor in IBD development [36,37,38]. The results of this study showed that neither DSS induction nor postbiotics significantly altered the alpha diversity of gut microbiota in mice. However, prophylactic gavage of *S. boulardii* significantly increased the Chao1 and ACE indices. This suggests that *S. boulardii* probiotics may tend to reshape the gut microecology during gut microbiota modulation, whereas postbiotics demonstrate greater mildness, making them more suitable for daily prevention.

Further analysis of the composition of the microbiota at the phylum and genus levels revealed that the phyla Firmicutes and Bacteroidota were the most abundant in the mouse gut. F/B is often considered a marker of microbial imbalance [39,40]. But in the DSS group of this study, the F/B ratio did not significantly decrease, a finding consistent with previous research [41,42]. In the prophylactic groups, the F/B ratio significantly increased in the BLD group, with the ratio in the PBLD group closest to that of the Control group. Through LefSe analysis, key gut microbiota genera associated with the preventive effects of *S. boulardii* and its postbiotics on UC were identified, including *Muribaculaceae*, *Akkermansia*, *Turicibacter*, and *Dubosiella*. *Muribaculaceae* is a beneficial gut bacterium and a major bacterial group in the mouse gut microbiota [43,44]. Our results showed that DSS induction decreased the relative abundance of *Muribaculaceae* in the mouse gut, and in the prophylactic groups, both BLD and DBLD groups further reduced the levels of *Muribaculaceae*, while only the PBLD group partially reversed this phenomenon. *Akkermansia* [45,46] and *Dubosiella* [47,48] are potentially beneficial probiotics for the gut, and their relative abundances changed significantly in the DBLD group, with *Akkermansia* showing a significant increase and *Dubosiella* showing a significant decrease. Additionally, *Turicibacter* can metabolize 5-hydroxytryptamine in the gut and is believed to be negatively correlated with butyrate levels [49,50]. This genus was not observed in the Control group, but DSS and *S. boulardii* treatment significantly increased its abundance, while both forms of postbiotics treatment reduced its abundance to a level similar to that of the Control group. These findings indicate that prophylactic gavage with *S. boulardii* may lead to significant changes and uncertainties at the genus level of the gut microbiota, warranting further research.

Postbiotics are traditionally considered to have numerous advantages over probiotics. This study confirms the role and advantages of postbiotics in the early prevention of UC. This understanding strengthens the rationale for using postbiotics and emphasizes their important role in promoting gut health and controlling UC. However, this study was primarily based on the observation of cellular structure and analysis of traditional indicators and has not delved into the specific mechanisms through which postbiotics regulate gut microbiota and alleviate inflammation, such as the regulation of key pathways or microbial metabolism. Additionally, the comparison of the effects of probiotics and postbiotics in this study was relatively limited, as the differences in their effects were not comprehensively assessed using multiple indicators and evidence. Future studies should employ more comprehensive indicators and deeper experimental designs to compare and evaluate the advantages and disadvantages of probiotics and postbiotics in clinical applications. While these issues still require further investigation, the findings of this study provide preliminary theoretical support for the application of probiotics and postbiotics in UC prevention, laying the foundation for future research. With the continued advancement of research on *S. boulardii* probiotics and postbiotics, it is believed that they will bring new breakthroughs in the prevention and treatment of UC in the future.

## 5. Conclusions

In conclusion, this study revealed the morphological characteristics of *S. boulardii* and its two forms of postbiotics through electron microscope structural characterization, providing intuitive morphological evidence for their functional mechanisms. Based on this finding, further research showed that pre-administration of *S. boulardii* and its postbiotics could regulate intestinal immune balance, enhance intestinal barrier function, and inhibit the expression of pro-inflammatory factors, significantly reducing the risk of DSS-induced colitis in mice and delaying disease progression. These results suggest that *S. boulardii* and its postbiotics have unique applications in disease prevention, offering new strategies and theoretical support for the early intervention of UC.

## Data Availability

The original contributions presented in the study are included in the article and Supplementary Materials, further inquiries can be directed to the corresponding author.

## Supplementary Materials

The following supporting information can be downloaded at: https://www.mdpi.com/article/10.3390/foods14071109/s1, Table S1: Disease activity index scoring criteria; Table S2: Primer sequences for real-time fluorescent quantitative PCR; Figure S1: Freeze-dried postbiotics (left) and spray-dried postbiotics (right); Figure S2: Relative abundance of verrucomicrobiota; Figure S3: Relative abundance of proteobacteria.
