# Supplementary material for: Pre-Administration of Saccharomyces boulardii-Derived Postbiotics Effectively Prevents Dextran Sulfate Sodium-Induced Colitis in Mice
Source: Foods. 2025 Mar 23;14(7):1109. doi: 10.3390/foods14071109 (PMC11988871; doi:10.3390/foods14071109)
Supplement: Supplementary file 1 [file foods-14-01109-s001.zip › foods-3491346-supplementary.pdf]

Supplementary

Table S1. Disease activity index scoring criteria

Score	Body weight loss (%)	Fecal traits	Hematochezia
0	≤0	Normal stools	Normal
1	1-5	Soft stools	Small amounts of blood-streaked feces
2	6-10	Loose stools	A certain amount of bloody feces
3	11-15	Semi-loose stools	Conspicuous blood-wrapped feces
4	>15	Watery stools	Visible rectal bleeding

The DAI calculating formulais as follows: DAI = 1/3 (Body weight loss score + Fecal traits score + Hematochezia score).

Table S2. Primer sequences for real-time fluorescent quantitative PCR

Target Gene	Nucleotide Sequence of Primer (5′ to 3′) Forward	Nucleotide Sequence of Primer (5′ to 3′) Reverse
GAPDH	ATGGTGAAGGTCGGTGTGAA	TTTGCCGTGAGTGGAGTCAT
IL- 1β	GTCGCTCAGGGTCACAAGAA	CCACACGTTGACAGCTAGGT
IL-6	GGAGCCCACCAAGAACGATA	GTCACCAGCATCAGTCCCAA
IL-10	AGAGAAGCATGGCCCAGAAA	ACACCTTGGTCTTGGAGCTT
TNF-α	AGATTCTTCCCTGAGGTGCA	ACCCCGGCCTTCCAAATAAA
Occludin	TTTCCTGCGGTGACTTCTCC	AAAACAGTGGTGGGGAACGT

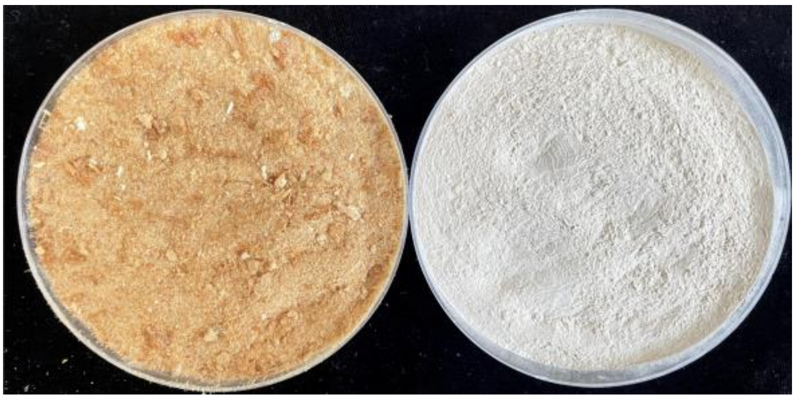

Figure S1. Freeze-dried postbiotics (left) and spray-dried postbiotics (right).

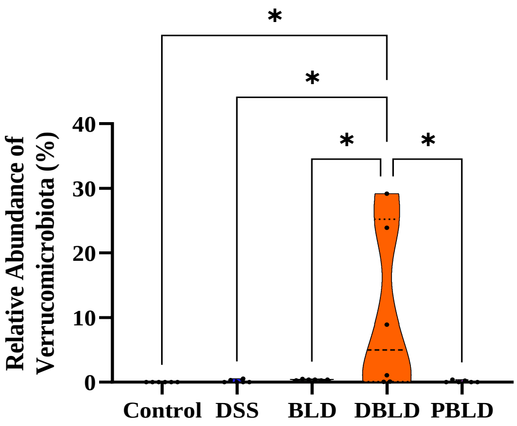

Figure S2. Relative abundance of verrucomicrobiota.

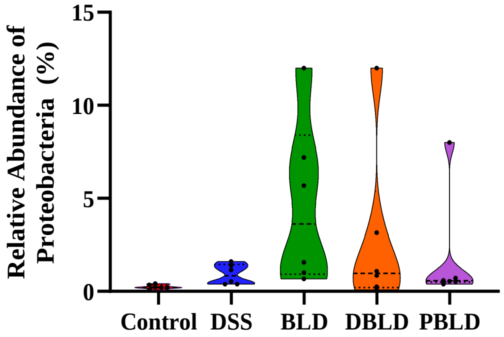

Figure S3. Relative abundance of proteobacteria.
